# Effect of dexmedetomidine on postoperative cognitive dysfunction in elderly patients undergoing orthopaedic surgery: study protocol for a randomized controlled trial

**DOI:** 10.1186/s13063-023-07110-9

**Published:** 2023-01-26

**Authors:** Weihong Zhao, Huanhuan Zhang, Jianli Li

**Affiliations:** 1 Department of Anesthesiology, Hebei General Hospital, Shijiazhuang, 050000 Hebei China; 2 Graduate School of Hebei Medical University, Shijiazhuang, 050000 Hebei China

**Keywords:** Dexmedetomidine, T helper 17 cell, Regulatory T cell, Postoperative cognitive dysfunction, Matrix metalloproteinase 9, Randomized controlled trial

## Abstract

**Aims:**

This trial aims to assess whether dexmedetomidine can reduce the incidence of postoperative cognitive dysfunction in elderly orthopaedic patients and explore the specific mechanism.

**Background:**

Postoperative cognitive dysfunction is a common complication after orthopaedic surgery that results in poor prognosis and increases the length of hospital stays and costs. Dexmedetomidine has been confirmed as a drug that can improve postoperative cognitive dysfunction in some studies. However, to date, the specific mechanism by which dexmedetomidine improves postoperative cognitive dysfunction is still elusive.

**Methods/design:**

A single-centre, prospective, double-blinded, randomized controlled trial will be conducted at Hebei General Hospital. Ninety-six elderly patients who undergo total hip or knee replacement will be studied in this trial and randomly divided into two groups. Patients in the experimental group will receive a loading dose of 0.5 μg/kg dexmedetomidine for 10 min and then a maintenance dose of 0.5 μg/kg/h dexmedetomidine until 30 min before the end of the operation, and patients in the control group will be infused with an equal volume of normal saline. The incidence of postoperative cognitive dysfunction will be the primary outcome. Changes in the balance of T helper 17 cell and regulatory T cell; the levels of matrix metalloproteinase 9, S-100β, IL-17A, and IL-10; perioperative complications; hospitalization duration; and intraoperative blood loss will be the secondary outcomes.

**Discussion:**

The consequences of this trial will show that dexmedetomidine can improve postoperative cognitive dysfunction in elderly orthopaedic patients, which may be related to the balance of T helper 17/regulatory T cells.

**Trial registration:**

Chinese Clinical Trial Registry ChiCTR2200055802. Registered on 20 January 2022

**Supplementary Information:**

The online version contains supplementary material available at 10.1186/s13063-023-07110-9.

## Background

Postoperative cognitive dysfunction (POCD) is defined as the patient’s cognitive dysfunction following surgery and anaesthesia, which mainly manifests as a decline in cognitive function and memory. The severely affected patients may experience a reduced capacity for social interaction and irreversible cognitive impairment [1]. It has been reported that the incidence of POCD in patients over 60 years old is 25 to 40% [2]. The incidence of POCD in patients undergoing lower extremity arthroplasty is 41–75% [3]. POCD, a severe postoperative complication, may result in increased time in bed and the risk of pulmonary infection. In addition, postoperative mortality will increase in severe cases [4]. Therefore, it is crucial to find a proper perioperative drug to prevent POCD.

Dexmedetomidine (DEX), a new α2 adrenergic receptor agonist, induces sedation, hypnosis and analgesia effects. In recent years, studies on DEX have been extensive, especially in research on neuroprotection [5]. There are increasing evidences that DEX plays a significant role in improving POCD [6–8], but the mechanism is uncertain. Studies have shown that the mechanism by which DEX improves POCD includes many factors, such as inhibition of the inflammatory response [9] and apoptosis [10]. However, due to the pleiotropic regulation of DEX, the molecular mechanism and specific regulatory pathway of the neuroprotective effects of DEX should be further confirmed.

Recently, there has been increasing concern about the effect of DEX on postoperative immune function [11–13]. T helper 17 (Th17) cells and regulatory T cells (Treg cell) are a subset of CD4_T cells, which have been shown to participate in the development of many inflammatory diseases, including systemic lupus erythematosus and multiple sclerosis (MS) [14, 15]. A study showed that vitamin D can improve POCD by regulating the Th17/Treg cell balance [16]. Another study reported that α7nAchR can improve POCD by regulating the Th17 response [17]. Additionally, a previous clinical study found that peripheral Th17/Treg imbalance was involved in the development of ischaemic stroke in elderly patients [18]. Another study reported that the Th17/Treg balance shifting to the Th17 cell played a role in the development of cognitive impairment in traumatic brain injury patients [19]. The above results revealed that the balance of Th17/Treg cells might play an important role in POCD. Moreover, previous literature showed that Treg cells can alleviate the damage to the blood-brain barrier induced by ischaemic stroke [20]. Matrix metalloproteinases (MMPs) are the main mediators that reflect the loss of blood-brain barrier (BBB) integrity, and they have been proven to be involved in the pathological process of many diseases such as AD and cerebral ischaemia reperfusion injury [21, 22]. Elevated MMP-9 levels in patients after surgery may lead to the occurrence of POCD [23]. However, whether DEX can improve POCD by alleviating the imbalance of Th17/Treg cells remains unknown.

The present study aims to verify whether DEX can improve POCD by regulating the balance of Th17/Treg cells in patients undergoing lower extremity arthroplasty. Health economic indicators (hospitalization duration and costs) will provide favourable evidence of DEX improving POCD in patients undergoing lower extremity arthroplasty. This trial will provide further trustworthy support for the intraoperative application of DEX to prevent the occurrence of POCD.

## Methods

This protocol was guided by the Standard Protocol Items: Recommendations for Interventional Trials (SPIRIT) 2013 Checklist (Additional file 1). The trial received ethics committee approval from Hebei General Hospital, China (ethics approval no.2019–48), and it was registered on ClinicalTrials.gov (No. ChiCTR2200055802). The trial registration dataset is presented in Additional file 2. Publications associated with the trial will be formatted according to the CONSORT Statement (Additional file 3).

### Trial design

This is a prospective, single-centre, double-blinded, randomized controlled trial. The study will be conducted at Hebei General Hospital for 24 months. All the participants will be randomized into two groups: the dexmedetomidine group (group D) and the control group (group C). Investigators will be screened according to the inclusion and exclusion criteria. Data collection will begin from the day before surgery to the end of follow-up (Table 1).

**Table 1:**
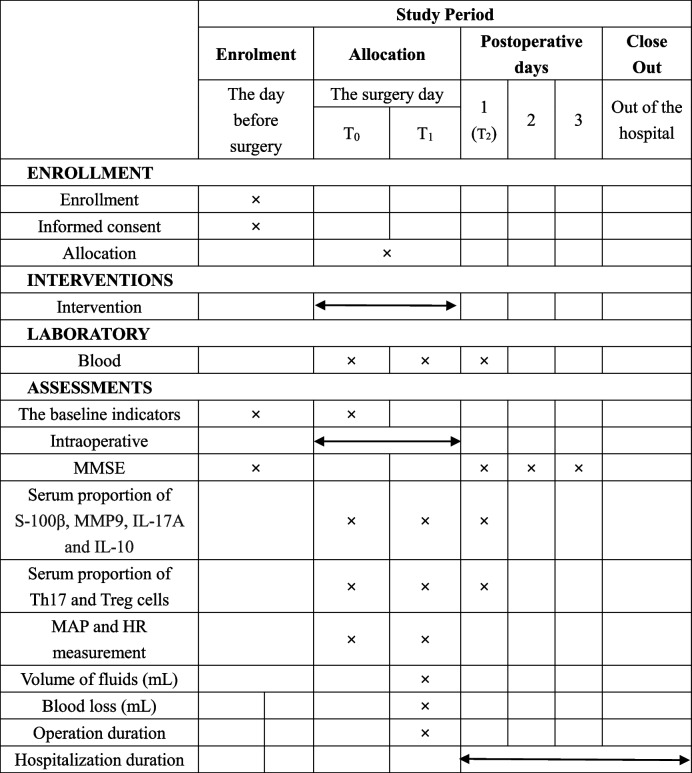
Standard Protocol Items: Recommendations for Interventional Trials (SPIRIT) Schedule for enrollment, interventions, and assessments

The purpose of this protocol is to design a high-quality randomized controlled trial with randomization of participants and blinded assessors to provide data about whether the application of DEX might reduce the incidence of POCD. However, the framework of this trial is not perfect. The included patients will have preoperative comorbidities, which may be associated with POCD. Stratified observation is desirable.

### Randomization and blinding

This study is planned to follow a double-blinded method. Anaesthesiologists will not be involved in collecting data, and participants will be unaware of the group assignments. Using the Microsoft Excel random number generator, the patients participating in the trial will be randomized into two groups at a ratio of 1:1. The envelopes containing the number will be opened after the patients enter the operating room. Based on the random number, participants will be allocated to either group D or group C with equal probability. The outcome assessors and statisticians will not be involved in intraoperative management and will conduct outcome assessments and statistical analyses separately.

### Study participants

**Fig. 1:**
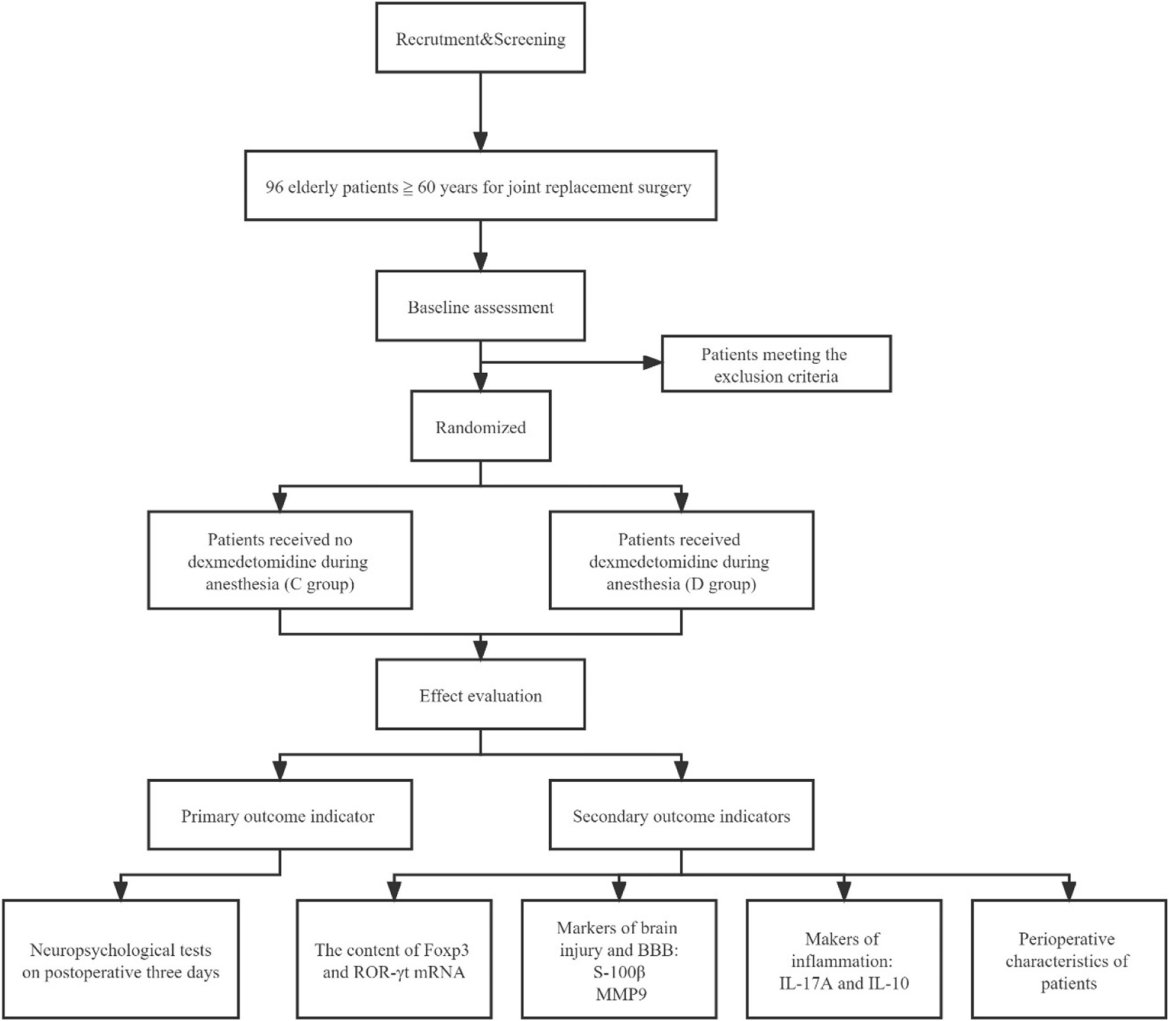
Consolidated Standards of Reporting Trials (CONSORT) diagram for this trial

We plan to recruit 96 patients who are scheduled to undergo lower extremity joint replacement surgery and determine eligible patients according to preoperative visits. The study process is shown in Fig. 1. An approved informed consent form will be signed by patients participating in this study (Additional file 4).

### Inclusion and exclusion criteria

The following are the inclusion criteria: (1) aged 60–80 years, regardless of gender; (2) undergoing hip or knee replacement surgery under combined spinal-epidural anaesthesia; (3) American Society of Anesthesiologists (ASA) classification I– III; (4) first language is Chinese; and (5) sign the informed consent to participate in the trial.

The following exclusion criteria will be used: (1) refusal to be included in this study, (2) a history of spinal surgery, (3) participation in other clinical trials in the past four weeks, (4) Mini-Mental State Examination scores < 21, (5) use of tranquillizers or antidepressants, (6) severe hearing and vision impairment, (7) contraindications to anaesthesia and (8) cancellation of the operation.

### Recruitment and informed consent

Elderly patients, who plan to undergo lower extremity joint replacement surgery, will be included in this study. This study received ethics committee approval from the Ethics Committee of Hebei General Hospital. Interested participants will receive a detailed explanation from the designated doctors and then receive an informed consent form for participating in the clinical trial and an informed consent form for collecting blood samples. Participants or their trustees or guardians will sign an informed consent and can withdraw at any time during the trial. Informed consent and the baseline data of patients will be obtained before allocation. Additionally, they can contact the doctors on this team if they have health problems. During the clinical trial, the researcher will report any serious adverse events to the director in charge at once, regardless of whether the event is related to the study drug.

### Intervention and anaesthesia regimen

Participants will not receive any special medications before surgery. Patients will undergo routine preoperative preparations. On the day of the operation, a peripheral vein passage will be established for patients. After entering the operating room, the ECG monitor will be routinely connected to measure the ECG, pulse oxygen saturation and noninvasive blood pressure. Patients will be given combined spinal-epidural anaesthesia by the same anaesthesiologist to ensure the operation. After asking the patients to turn to the lateral position, we will select the puncture site between lumbar vertebrae 2 and 3. After confirmation of a successful puncture, 3 ml of 0.5% bupivacaine will be injected into the subarachnoid space; afterwards, an epidural catheter will be placed. If the level of intraoperative sensory block is inadequate, 3–5 ml of 2% lidocaine will be added to the epidural catheter to maintain the sensory block level below T10 according to the surgical process and clinical manifestations of the patient during the surgery. At the end of the surgery, the epidural catheter will be removed. Femoral nerve blocks will be used for postoperative analgesia.

In the experiment group (group D), patients will be intravenously infused with a loading dose of 0.5 μg/kg dexmedetomidine using a micropump for 10 min and then a maintenance dose of 0.5 μg/kg/h until 30 min before the end of the operation.

In the control group (group C), patients will be intravenously infused with a loading dose of 0.5 μg/kg normal saline using a micropump for 10 min and then a maintenance dose of 0.5 μg/kg/h until 30 min before the end of the operation.

If the patient’s blood pressure drops to more than 20% of the basal blood pressure, 6 mg of ephedrine will be given intravenously each time, and if bradycardia occurs (25% reduction from baseline), 0.5 mg atropine will be injected. Patients with SpO_2_ < 90% requiring oxygen therapy, severe complications from preoperative comorbidities or failure of spinal anaesthesia will be excluded from this study.

### Interventions, modifications, adherence and concomitant care

The assigned intervention will be stopped only in response to the participant’s request. We plan no modification of the interventions during the trial. Adherence to interventions mainly refers to patient self-management adherence. No concomitant care or interventions are permitted during this trial.

### Outcome assessment

The primary endpoint will be the incidence of POCD within 3 days postoperatively in orthopaedic patients. POCD will be evaluated using the Mini-Mental State Examination (MMSE), which is widely used in clinical and research settings to measure cognitive impairment [24]. The investigators will evaluate cognitive impairment 1 day before surgery, as well as on the first and third postoperative days. A 2-point reduction in MMSE scores after surgery was regarded as POCD [25].

The secondary endpoints will include the specific biochemical markers of brain injury in plasma, for example, S-100β protein levels, a related indicator of BBB disruption such as MMP9, and inflammatory factors, such as IL-17A and IL-10, will be detected by ELISA. qRT-PCR will be used to detect RORγt/Foxp3 mRNA to reflect the Th17/Treg balance. We will collect the 5 ml of the patients’ peripheral blood before the operation, at the end of the operation and the first day after the operation and save it for follow-up testing. In addition, secondary indicators will also include the operation duration, intraoperative blood loss, positioning of the patient, total amount of intraoperative epidural lidocaine, length of hospital stay and general conditions of the patients, such as age, sex, weight, education level and whether the patient has complications such as hypertension and diabetes before surgery. The occurrence of other events such as postoperative bleeding, infection and reoperation will be recorded simultaneously. The secondary results will be evaluated by the medical team. The length of hospital stay is calculated based on the number of days from the date of surgery to the date of discharge. The criteria for discharge will include stable vital signs, and no other complications after surgery.

### Sample size

The PASS 15.0 software will be used to calculate the sample size. According to the previous study [3], we hypothesized that the incidence of POCD in elderly patients undergoing lower extremity arthroplasty is 45%. In our pilot study, a total of 15 patients were included in group D, and 3 patients were diagnosed with POCD. On this basis, a sample size of 41 patients in each group will ensure a power of 0.80 to detect differences at the 0.05 level of significance. Considering the dropout rate, 96 patients will be included.

### Data management

The data of all patients participating in this trial will be recorded in the case report form (CRF) of each patient. The patient’s participation in this trial must be recorded in the CRF, including the study number, subject number, subject information, informed consent and date of each visit. Source data should be archived in accordance with the GCP guidelines. Based on the sponsor’s standard operating procedures, the data manager will be responsible for data processing and checking the completeness and accuracy of data input. The database will be locked only after the quality assurance process is completed.

### Statistical analysis

SPSS 24.0 (*SPSS Inc*.) will be used for statistical analysis. For variables not normally distributed, continuous data will be represented by the mean ± standard deviation (SD) or median (interquartile range (IQR)), and parametric or nonparametric *t* tests will be used for comparisons. Normally distributed data will be analysed using the Shapiro-Wilk test. Normally distributed data will be compared among the two groups using one-way *ANOVA* with *LSD-t* as the post hoc test. Categorical variables will be presented as proportions and analysed using the chi-square (or Fisher’s exact) test. Two-sided *p* values less than 0.05 will be considered statistically significant. Participants with missing primary or secondary outcome data will be excluded. Patients excluded after randomization will be described descriptively.

### Data collection methods and monitoring

Statistical professionals are responsible for developing statistical analysis plans in consultation with the principal investigators, establishing databases and using the SPSS statistical analysis system to perform the statistics. Comprehensive efficacy analysis was conducted according to the programme set and efficacy indicators such as the complete analysis set, demographics and other baseline characteristics. The results will be available to the public after publication.

### Composition of the coordinating centre and trial steering committee

The coordinating centre consists of clinicians from the Department of Anaesthesiology and the Department of Orthopedics of Hebei General Hospital. The principal investigators will supervise this trial and be responsible for the medical responsibility of patients. The data manager will collect the data carefully. The study coordinator will register this trial and coordinate study visits and safety reporting. The surgeons will evaluate patients’ postoperative recovery. The study team will meet once a week. There is no trial steering committee (TSC) for this trial.

### Composition of the data monitoring committee

A data monitoring committee will not be designed for this trial. However, the principal investigator will regularly monitor the participants’ safety, the accuracy of data, and the conduct of the trial.

### Safety evaluation

Adverse events (AEs) refer to the appearance or progression of any symptoms of discomfort, syndromes or diseases that affect the health of the patients during clinical trials. Any indication of disease and/or organ toxicity and abnormalities requiring immediate treatment in clinical trials are considered AEs. In the process of research, the clinical manifestations, discovery time, severity, duration, measures taken, treatment results and any potential relationship between the drug and the AEs will be recorded by the study coordinator in detail.

Once AEs occur, the observing doctor should decide whether to stop the observation according to the situation and report the AEs to the ethics committee and the principal investigators. All adverse events should be tracked and recorded in detail until the individual’s problem is properly resolved or stabilized. Treatment will be started for patients with severe adverse events, and compensation will be completed as soon as possible.

### Validity and reliability

All data will be checked immediately after collection, and problems will be corrected by the researcher. All suspicious data will be checked with reference to the original data sources. Related statistical assumptions will be tested before conducting data analyses. All the variables will be compared between the groups at the beginning to guarantee equivalence. If there are any differences between the two groups at baseline for outcome variables, they will be controlled statistically in the analyses.

## Discussion

POCD, a common postoperative complication, refers to a decline in nervous system function, which might eventually lead to permanent brain damage [26]. As mentioned earlier, a higher incidence of POCD has been confirmed in patients undergoing lower extremity joint replacement surgery, but it remains hard to treat effectively. Researchers have proposed various hypotheses to reveal the mechanisms of POCD, including neuroinflammation, oxidative stress, autophagy disorder, impaired synaptic function and a lack of neurotrophic support [27]. However, the specific mechanism of POCD is still ambiguous.

Based on previous studies, surgery activates the host’s innate immune system, leading to peripheral inflammation [28, 29]. Therefore, we note that the changes in patients’ immune function due to surgery and anaesthesia may be an important factor affecting POCD. Treg cells, a special subset of T cells, play an important role in suppressing inflammation and inhibiting autoimmune processes [30] and appear to be an endogenous protective mechanism against brain inflammation after ischaemic stroke [20]. Th17 cells, also a subset of CD4 T cells, are involved in the pathological process of various central inflammatory diseases [31, 32]. A study showed that Th17 cells played a major role in the pathogenesis of neurodegenerative diseases [33]. According to the above studies, the imbalance of Th17/Treg cells may activate the inflammatory response and promote the release of inflammatory factors, which results in the occurrence and development of POCD.

Impairment of cognitive function may also be attributed to disruption of the BBB [34, 35]. The major components of the BBB include endothelial cells, astrocytes, pericytes and the extracellular matrix (ECM), which provide structural and functional support for the BBB. The ECM, one of the important components of the BBB, is destroyed by MMPs. MMP-9, an indicator of the damage of BBB, plays a vital role in the disruption of the BBB, neutrophil infiltration and brain injury after ischaemia stroke [36]. After the breakdown of the BBB, a large number of immune cells enter the brain, thus beginning a vicious cycle that causes long-term brain damage and disruption of neurological function.

Fortunately, many reports showed that DEX might improve postoperative immune function in patients. Researchers have reported that DEX can maintain the Th1/Th2 balance to protect immune function [12]. According to a recent study [13], DEX could regulate the CD4/CD8 balance in patients undergoing hip arthroplasty to protect immune function. Additionally, one study confirmed that DEX alleviated cognitive impairment by modulating Th1/Th2/Th17 polarization and reducing disruption of the BBB in a mouse experimental sepsis model [37]. Another study reported that Treg cells can alleviate the loss of BBB integrity after ischaemia stroke [20]. Here, we speculate that DEX could improve POCD by regulating the balance of Th17/Treg cells, which is associated with the protection of the BBB.

### Limitations

This study has some limitations. First, clinical trials are conducted in a single centre with a relatively small sample size, and there may be a bias in the yield of enrolled participants. Second, the observation time of this study is short, and the cognitive function of the patients will be observed within 3 days after surgery. The long-term effect of DEX on the cognitive function of elderly orthopaedic patients remains to be further confirmed.

## Conclusion

In general, this study may provide evidence of the efficacy and safety of DEX on POCD in elderly orthopaedic patients, and the underlying mechanism may be related to the balance of Th17/Treg cells.

## Trial status

The trial is ongoing and recruiting participants. This study protocol is version 1 made on February 1, 2022. Recruitment commenced in March 2022 at Hebei General Hospital and is expected to be completed in April 2023.

## Supplementary Information

**Additional file 1.** SPIRIT Checklist for Trials.

**Additional file 2.** All items from the World Health Organization Trial Registration Data Set.

**Additional file 3.** CONSORT 2010 checklist of information to include when reporting a randomised trial*.

**Additional file 4.** Informed Consent Form.

## Data Availability

The datasets used and/or analysed after completing the current study will be available from the corresponding author by reasonable request.

## Abbreviations

AEs: Adverse events
BBB: Blood-brain barrier
CRF: Case report forms
DEX: Dexmedetomidine
ECM: Extracellular matrix
MMP9: Matrix metalloproteinase 9
MMSE: Mini-Mental State Examination
POCD: Postoperative cognitive dysfunction
SPIRIT: Standard Protocol Items: Recommendations for Interventional Trials
