# Supplementary material for: Effect of dexmedetomidine on postoperative cognitive dysfunction in elderly patients undergoing orthopaedic surgery: study protocol for a randomized controlled trial
Source: Trials. 2023 Jan 26;24:62. doi: 10.1186/s13063-023-07110-9 (PMC9881307; doi:10.1186/s13063-023-07110-9)
Supplement: Supplementary file 2 — Additional file 2. All items from the World Health Organization Trial Registration Data Set. [file 13063_2023_7110_MOESM2_ESM.doc]

**2b: All items from the World Health Organization Trial Registration Data Set.**

| **Data category** | **Information** |
| --- | --- |
| Primary registry and trial identifying number | Chinese Clinical Trial Registry. ChiCTR2200055802 |
| Date of registration in primary registry | 20 January 2022. |
| Secondary identifying numbers | NA |
| Source(s) of monetary or material support | Key Research and Development Program of Hebei Province (Grant No. 19277714D) |
| Primary sponsor | NA |
| Secondary sponsor(s) | NA |
| Contact for public queries | Jianli Li, MD, PhD; E-mail: [hblijianli@163.com](mailto:hblijianli@163.com) |
| Contact for scientific queries | Jianli Li, MD, PhD  Hebei General Hospital, China |
| Public title | Effects of dexmedetomidine on postoperative cognitive dysfunction in patients undergoing lower extremity orthopedic surgery |
| Scientific title | Effect of dexmedetomidine on postoperative cognitive dysfunction in patients undergoing lower extremity orthopedic surgery: the association with Th17 and Treg cells |
| Countries of recruitment | China |
| Health condition(s) or problem(s) studied | Dexmedetomidine (DEX), anesthesia management, orthopedic surgery, postoperative cognitive dysfunction (POCD) |
| Intervention(s) | The application of DEX at perioperative phases; |
| Key inclusion and exclusion criteria | Inclusion criteria: (1) undergoing lower extremity orthopedic surgery under epidural anesthesia; (2) aged 60-80 years, regardless of gender; (3) American Society of Anesthesiologists (ASA) classification I– III; (4) sign the informed consent to participate in the trial.  Exclusion criteria: (1) refuse to be included in this study; (2) a history of spinal surgery; (3) Mini-Mental State Examination scores＜21; (4) take tranquillisers or antidepressants; (5) severe hearing and vision impairment; |
| Study type | Single-center, Randomized, Parallel controlled trial |
| Date of first enrolment | January 2022 |
| Target sample size | 70 |
| Recruitment status | Recruiting |
| Primary outcome | The Incidence of POCD |
| Key secondary outcomes | The changes in the balance of T helper 17 (Th17) cell and regulatory T (Treg) cell; The content of matrix metalloproteinase 9 (MMP9) and S-100β; Postoperative complications; |

**3. Date and version identifier**

Revision Chronology:

| 2022- January-20: | Original |
| --- | --- |
