# Supplementary material for: Effect of dexmedetomidine on postoperative cognitive dysfunction in elderly patients undergoing orthopaedic surgery: study protocol for a randomized controlled trial
Source: Trials. 2023 Jan 26;24:62. doi: 10.1186/s13063-023-07110-9 (PMC9881307; doi:10.1186/s13063-023-07110-9)
Supplement: Supplementary file 4 — Additional file 4. Informed Consent Form. [file 13063_2023_7110_MOESM4_ESM.docx]

右美托嘧啶对老年骨科手术患者术后认知功能障碍的影响：一项随机对照临床研究

知情同意书

Informed Consent Form

知情同意书版本号： Version 1.0 临床研究单位： _______________________________

主要研究者： _______________________________

联 系 电 话： _______________________________

右美托嘧啶对老年骨科手术患者术后认知功能障碍的影响

受试者知情同意书

我已收到并详细阅读了针药复合麻醉在腹部手术的应用及机体保护效应的多 中心，随机，对照，盲法的临床研究的受试者须知。我已了解这是一项临床试验，我的医生已经向我详细介绍了该临床试验的目的、方法、可能出现的不良反应以及我的风险和受益，并对有关问题给予了解答。我的医生还向我详细解释了本次试验我需要进行的配合。我在充分了解须知全部内容以及参加试验可能带来的利弊后，自愿参加并认真完成本试验。我理解：

1．尽管研究记录被保密，但各级食品药品监督管理部门、伦理委员会及申办者指派的稽查员和监查人员都有可能接触到我的个人资料，以上人员有责任为我的 个人情况保密。

2．我是本研究的受试者，我将遵守本研究方案和受试者须知的要求，完成本试验。

3．如果在试验过程中出现任何不良事件，我都会立即通知我的医生。

4．在研究过程的任何阶段，我有权退出，而不会受到歧视和报复。

5．我理解参加此项研究可能的受益和风险，我愿意为医学科学的进步做出奉献。

6．我自愿参加本试验，并与研究人员充分合作。

7. 我将获得一份由双方签名并注明日期的知情同意书副本。

受试者签名 日期 年 月 日

我已给予受试者足够的时间就受试者须知、知情同意书和试验方案等提出问题，并尽我所能给予了回答。

研究者签名 日期 年 月 日

联系方式：电话：

地址：

**Effect of** **Dexmedetomidine on postoperative cognitive dysfunction in elderly patients undergoing** **orthopedic surgery:** **a randomized controlled trial**

Informed Consent Form

**Version** **Number** **of** **Informed** **Consent**： Version 1.0

**Clinical** **Research** **Unit**：

**Major** **Researcher**：

**Telephone** **number.**：

Effect of Dexmedetomidine on postoperative cognitive dysfunction in elderly patients undergoing orthopedic surgery

Informed Consent Form

I have received and read in detail the instructions for participants in a multicenter, randomized, controlled, blind clinical study of the effect of Dexmedetomidine on postoperative cognitive dysfunction in elderly patients undergoing orthopedic surgery. I have learned that this is a clinical trial, and my doctor has given me a detailed description of the purpose, method, possible adverse reactions, and my risks and benefits of the clinical trial, and answered the relevant questions. My doctor also explained to me in detail the cooperation, I needed in this experiment. After fully understanding the contents of the instructions and the pros and cons of participating in the experiment, I volunteered to participate in and conscientiously completed the experiment. I understand:

1. Although the research records are kept confidential, the inspectors and inspectors appointed by food and drug regulatory authorities, ethics committees and bidders at all levels may have access to my personal data. The above persons are responsible for keeping my personal information confidential.

2. I am the subject of this study. I will complete this experiment in accordance with the requirements of the research program and the participants' instructions.

3. If any adverse events occur during the experiment, I will inform my doctor immediately.

4. At any stage of the research process, I have the right to withdraw without discrimination or retaliation.

5. I understand the potential benefits and risks of participating in this research. I am willing to contribute to the progress of medical science.

6. I volunteered to participate in the experiment and cooperated fully with the researchers.

7. I will get a copy of the informed consent signed and dated by both parties.

Subject signature ___________ Date____________

I have given the subjects enough time to ask questions about the subject's

instructions, informed consent and test plan, and to answer their questions to the best of

my ability.

Researcher's signature__________ Date: ______

Contact information： Tel：______

Address:

_____________________________
